# Copper as diagnostic marker of cancers

**DOI:** 10.1186/1897-4287-13-S2-A7

**Published:** 2015-11-26

**Authors:** Magdalena Muszyńska, Wojciech Marciniak, Katarzyna Jaworska-Bieniek, Katarzyna Kaczmarek, Grzegorz Sukiennicki, Marcin Lener, Katarzyna Durda, Tomasz Huzarski, Tomasz Byrski, Jacek Gronwald, Oleg Oszurek, Cezary Cybulski, Tadeusz Dębniak, Antoni Morawski, Anna Jakubowska, Jan Lubiński

**Affiliations:** 1 Department of Genetics and Pathology, International Hereditary Cancer Center, Pomeranian Medical University, Szczecin, Poland; 2 Read - Gene, S.A., Grzepnica, Poland

## 

The study was conducted to determine if serum copper level could be a useful marker for selection for control examinations and if serum copper level is a risk factor in developing cancer.

Copper was quantitatively measured in diluted serum samples by inductively coupled plasma mass spectrometry (ICP-MS) using mass spectrometer (Elan DRC-e, PerkinElmer) in standard mode. In our study, there were two independent groups of patients examined. In the first, retrospective model, there were patients diagnosed with prostate cancer (n = 166) and laryngeal cancer (n = 123) matched with healthy controls. This study showed that serum copper level above 1250 µg/l may be a useful marker for laryngeal examination, but is not a useful marker for prostate cancer early detection. In the second, prospective model, there were patents diagnosed with breast cancer (n = 42) matched with unaffected controls. Serum from breast cancer patients was collected 3 - 41 months before cancer diagnosis. This part of study showed that there is a tendency that breast cancer risk is about two times lower when copper serum level is in range between 1035 - 1311 µg/l. Further investigations are needed.

